# Issues Associated With the Management and Governance of Sensor Data and Information to Assist Aging in Place: Focus Group Study With Health Care Professionals

**DOI:** 10.2196/24157

**Published:** 2020-12-02

**Authors:** Inga Hunter, Phoebe Elers, Caroline Lockhart, Hans Guesgen, Amardeep Singh, Dick Whiddett

**Affiliations:** 1 School of Management Massey University Palmerston North New Zealand; 2 School of Communication, Journalism and Marketing Massey University Palmerston North New Zealand; 3 School of Fundamental Sciences Massey University Palmerston North New Zealand

**Keywords:** smart home, home monitoring technology, aging in place, information governance, information management, older people, support network, aging, elderly health

## Abstract

**Background:**

Smart home and telemonitoring technologies have often been suggested to assist health care workers in supporting older people to age in place. However, there is limited research examining diverse information needs of different groups of health care workers and their access to appropriate information technologies.

**Objective:**

The aim of this study was to investigate the issues associated with using technologies that connect older people to their health care providers to support aging in place and enhance older people’s health and well-being.

**Methods:**

Seven focus group discussions were conducted comprising 44 health care professionals who provided clinic-based or in-home services to community-dwelling older people. Participants were asked about their information needs and how technology could help them support older people to age in place. The recordings of the sessions were transcribed and thematically analyzed.

**Results:**

The perspectives varied between the respondents who worked in primary care clinics and those who worked in community-based services. Three overarching themes were identified. The first theme was “access to technology and systems,” which examined the different levels of technology in use and the problems that various groups of health care professionals had in accessing information about their patients. Primary care professionals had access to good internal information systems but they experienced poor integration with other health care providers. The community-based teams had poor access to technology. The second theme was “collecting and sharing of information,” which focused on how technology might be used to provide them with more information about their patients. Primary care teams were interested in telemonitoring for specific clinical indicators but they wanted the information to be preprocessed. Community-based teams were more concerned about gaining information on the patients’ social environment. The third theme was that all respondents identified similar “barriers to uptake”: cost and funding issues, usability of systems by older people, and information security and privacy concerns.

**Conclusions:**

The participants perceived the potential benefits of technologies, but they were concerned that the information they received should be preprocessed and integrated with current information systems and tailored to the older people’s unique and changing situations. Several management and governance issues were identified, which needed to be resolved to enable the widespread integration of these technologies into the health care system. The disconnected nature of the current information architecture means that there is no clear way for sensor data from telemonitoring and smart home devices to be integrated with other patient information. Furthermore, cost, privacy, security, and usability barriers also need to be resolved. This study highlights the importance and the complexity of management and governance of systems to collect and disseminate such information. Further research into the requirements of all stakeholder groups and how the information can be processed and disseminated is required.

## Introduction

Like in other developed nations, life expectancy in New Zealand is increasing, which is resulting in an aging population [1]. It is estimated that the proportion of the New Zealand population aged 65 years and older will increase to 21%-26% in 2043 and 24%-33% in 2068 [2]. As older people have higher rates of chronic conditions and disabilities that require regular support [3], this can place an increased demand on health care services. An option favored by policymakers [4] and older people [5] is to support individuals to remain in their own homes for longer and avoid residential care, which is known as “aging in place.”

Various technologies have been used to support older people as they age in place, including home-monitoring devices [6], purpose-built smart homes [7], intelligent cognitive assistants [8], and web-based health information resources [9]. However, many technologies have the limitation of only treating 1 condition in isolation, rather than the older person as a whole, who may be dealing with a range of health issues and receiving services from a range of formal health care providers.

Many older people also rely on informal support networks of friends, neighbors, and family members who provide ongoing practical and emotional support such as personal care, household work assistance, company, and emotional assistance. Such informal support networks would also benefit from access to information from these support technologies; Fischer et al [10] reports the following about informal support networks, “Tools for the elderly should consider the whole care network and take into account who will be using the tool, who has access to what information, and how these factors may change over time.”

This paper presents some of the findings from an exploratory project that investigates how technologies that connect older people to their informal and formal support networks could assist aging in place and enhance older people’s health and well-being. In the initial phase of the project, we explored the requirements of the many stakeholder groups involved; in the second phase, these requirements informed the design of prototype technology [11], which has been used and evaluated by older people and their informal support networks [12].

Stakeholder requirements were identified in 3 ways; in each case, issues of information governance were found to be very important. First, we solicited the perspectives of experts in telemedicine and health informatics by organizing a workshop [13]; this research highlighted the importance of data integration, security, and control. Second, we conducted in-depth interviews with older people and their self-identified informal support networks [14]; these interviews emphasized the importance of information security and access controls. Finally, in this paper, we present the findings related to the needs of the formal support network from 7 focus groups, which consisted of 44 health care professionals working with community-dwelling older people. The focus groups explored the types and sources of patient-related information the various groups needed to support their work, how they accessed the information, and how telemedicine or smart home technologies might provide them with additional useful information about their patients. The findings of this study highlight the different resources and information requirements of the many stakeholder groups who may be involved, thereby showing that information governance issues are important.

The work reported here adds to the scholarly body of knowledge by examining the various and differing information requirements and technological capabilities of different groups of health care professionals who could benefit from using home-monitoring technologies to support the health of older people living in their own homes. The qualitative nature of the research means that care should be taken when generalizing the findings beyond the study locations; however, this study is valuable because it highlights the need to address issues of information access and governance, which have been largely neglected in related health informatics research to date.

## Methods

### Focus Groups

Seven face-to-face focus groups were conducted with participants employed at health care organizations that provide support for older people. The focus groups were conducted at the respective organizations where the participants were employed during normal working hours. On average, each focus group lasted for 1 hour. The study procedures were approved by the Massey University Human Ethics Committee (SAO 16/65).

### Recruitment

Participants were recruited through purposive convenience sampling, which aimed to gather a range of contrasting perspectives. We contacted a range of health care organizations that provide various support services for older people in the Manawatū region in New Zealand, requesting their cooperation and that information about the study be distributed to staff. The focus groups were voluntary, and interested participants chose to attend and participate. They did not receive financial incentives for participation; 44 participants took part in the study. They were predominantly women and their average age was 46.6 years. Further information about the focus groups is outlined in Table 1.

Focus groups 1 to 4 were composed of employees of the local public health system. These different professional teams, physiotherapists, occupational therapists, social workers, and nurses were based at the local hospital but provided services to community-dwelling patients in their homes. Primarily, they provide ongoing services to people who have been discharged from the hospital and to clients who have been referred from primary care practices. Each of the other 3 focus groups consisted of team members from primary care practices, which were based in clinics within the community. These groups primarily provide services at their clinics with limited community-based services. The sample size was appropriate because while there was some variation in the perspectives between the hospital-based teams and the primary care teams, there were repeated commonalities across the focus groups, whereby researchers deemed that data saturation was met.

**Table 1:** Composition of the focus groups.

Characteristics	Focus group 1 (n=6)	Focus group 2 (n=6)	Focus group 3 (n=5)	Focus group 4 (n=5)	Focus group 5 (n=8)	Focus group 6 (n=9)	Focus group 7 (n=5)
Organization type	Public health system	Public health system	Public health system	Public health system	Primary care practice	Primary care practice	Primary care practice
Participants’ occupations	3 physiotherapists 2 occupational therapists 1 clinical coordinator	6 social workers	5 social workers	5 nurses	1 general practitioner 4 nurses 1 clinical director 1 business manager 1 clerk	2 general practitioner 1 urgent care doctor 2 nurses 1 social worker 1 clerk 2 directors	1 social worker 2 geriatricians 2 nurses

### Focus Group Design and Content

Focus groups were used because they are an efficient method for obtaining data from multiple participants [15], and they can stimulate in-depth discussion around key ideas [16]. The flexible nature of the focus groups means that researchers can probe and clarify meanings that may be implied or unclear [17]. Conducting focus groups involves the negotiation of complex power and social dynamics between the researcher and participants and within the participant group in response to the research setting and wider context [18]. For instance, within health organizations, there are likely to be existing hierarchical and professional structures. Although this can never be entirely overcome in a focus group setting, we tried to address this by listening to what was being said, by whom, and under what conditions, and asking and probing for different participants to share their opinions.

Focus group questions were developed to address the project’s purpose and increase consistency across sessions. The development of these questions was guided by a workshop conducted with participants attending a health informatics conference [13], which provided another lens of the research procedures to help reduce an unconscious bias in the research design. Each focus group began with the distribution of participant information sheets and consent forms, followed by an introduction about the project and the use of home-monitoring technology and information and communication technologies connecting older people to their informal and formal support networks to assist aging in place. Participants were then asked about their information needs, what information should be collected and transferred, who should receive this information, as well as the potential ethical concerns.

### Analysis

The focus groups were transcribed and thematically analyzed [19] inductively in NVivo Version 11.0 (QSR International). Owing to the nature of the focus groups, individual participants were not identified. Thematic analysis is widely used within the social and health sciences [20] as a tool to examine “repeated patterns of meaning” [18] or as a way of identifying and making sense of commonalities within data sets [19]. The coding and theme development were inductive, using an iterative process that involved reading and rereading the data sets to establish initial codes that covered the key ideas discussed and then combining similar codes under the themes. Following this process, the themes were reviewed alongside the original data set as a way of checking that the data set was correctly represented and that important data were not missed. The analysis was undertaken by a primary researcher, which was discussed within the research team throughout the analysis and cross-checked by a second researcher.

## Results

### Overview of the Findings

The New Zealand public health care system has a bifurcated structure and complex funding structures, which complicates the sharing of information systems and of patient information. The government funds health care on a regional basis through several district health boards, which directly run their local hospital-based services and some community services that are based at the hospital, such as those delivered by the members of focus groups 1-4. The services that are delivered directly by the district health boards are totally tax-payer funded and free at point of care. The delivery of local primary care services is essentially outsourced by the district health boards to several independent primary care practices, such as those observed in focus groups 5-7. Most of the services they provide are heavily subsidized by the district health boards but most patients are required to make some additional payment for the services they receive. The primary care practices each operate their own sophisticated patient management systems.

The analysis of the focus groups identified 3 overarching themes: (1) access to technology and systems, (2) collecting and sharing of information, and (3) barriers to uptake. Each theme has several subthemes, which are summarized in Table 2 and described below. An important observation from our analysis of the different focus groups is the contrast in the facilities and the perspectives between the members of the teams working in the community within the public health system and those of the primary care teams.

**Table 2:** Summary of the major themes and perspectives of the respondent groups.

Themes, subthemes		Perspectives	
		Hospital-based teams, focus groups 1-4	Primary care teams, focus groups 5-7
**Access to technology and systems**			
	Technologies used	Multiple fragmented systems, paper-based notes	Good integrated internal system
	Major limitations	No access to primary care systems, no mobile access to systems	Poor integration with hospital systems
**Collecting and sharing of information**			
	Additional information required	Information on background and social environment	Less interest in social information
	Interest in telemonitoring	Monitoring of falls highly desirable, little desire for clinical monitoring	Monitoring of falls highly desirable, interest in telemonitoring specific clinical indicators, but preprocessing of data required
**Barriers to uptake**			
	Usability	Concerns about dexterity requirements and cognitive decline of patients	Concerns about dexterity requirements and cognitive decline of patients
	Cost	Concerns about system funding and cost to patients	Concerns about system funding and cost to patients
	Security, privacy, and confidentiality	Concerns about patient privacy and possible breeches of confidentiality	Concerns about patient privacy and possible breeches of confidentiality

### Theme 1: Access to Technology and Systems

Despite many years of development and implementation of integrated health information systems, many respondents complained about the way patient information remains fragmented within multiple systems. There are multiple systems within different parts of the hospital, which are not universally accessible to the staff members of the different focus groups who might need the information, and this leads to frustration and inefficiencies.

…Probably the easiest way is to say multiple computer systems. And not everybody has access to the same information.

Focus group 3

While the hospital-based teams have some access to hospital computer systems, their own on-going notes are paper-based and only a final summary is shared to an electronic system.

…The referrals get processed into the computer but then that’s printed off…all our records are paper-based.

Focus group 4

…If I have finished seeing Mrs. Smith, we do a discharge summary and type that up and it goes on to a clinical portal. So, discharge summaries or clinical letters can go onto their file but the actual running notes of “I saw someone and provided something” is all written by hand.

Focus group 1

This leads to problems coordinating care within and across teams.

…I mean there are just lots of people collecting data and then keeping it to themselves essentially and if you want to get as much data on this person as you can, you have to physically run around grabbing files and saying what are your thoughts and what have you noticed, rather than actually having it as a central accessible thing.

Focus group 1

Another issue for the hospital-based staff is the lack of access to information from the primary care clinics, which operate their own systems.

…One big gap is that we don't have access to the GP records. We need to physically ring, have a chat. We can't just login, you know just to kind of get an idea.

Focus group 3

In contrast to the hospital-based teams, the primary care teams run their own sophisticated electronic systems, but they also complained about the lack of integration with other service providers.

…Pretty much everything from the hospital in terms of what information you receive, district nursing discharges, physio, everything we get… comes via fax or by post, we have to scan it into the notes.

Focus group 5

All groups also raised the issue of the difficulty of obtaining a complete picture of the patients and the presence of gaps in information from other government organizations or from nongovernment service providers. For example, many clients paid for personal alarm systems, which could summon paramedical assistance when needed, for example, in case of a fall, but these service providers tended not to share their patient information with the public health system.

…Older patients see so many other providers and nongovernment service providers and social services that we don’t ever get any feedback about.

Focus group 5

### Theme 2: Collecting and Sharing of Information

As noted in Theme 1, all respondents complained of the difficulties of locating patient information, which could be distributed across multiple disconnected systems, and all felt that better integrated systems would be beneficial for everyone. Focus groups also explored the additional information they would like to have available, which was not currently being collected. Some of this information could be collected by remote monitoring technologies but some information would need to be collected and shared by practitioners after their visits. Again, the perspective sometimes varied depending on whether the focus group participants worked in the community or in the primary care practice. Community practitioners, in particular, wanted more information about patients’ background data and social environment before they made a home visit.

…[You] go and see Mrs. Smith, she has no arms and legs, isn't English speaking, has a pressure ulcer, falls over 10 times a day, but nothing is written on the referral.

Focus group 1

…You don’t really know what you’re walking into. You could be going into an 80-year-old at home with no fire lit, no food, or you could be going to the opposite.

Focus group 4

This could also be a matter of personal safety.

…So it would be really nice for us to see, right, alcoholic, maybe we should take somebody else with us on this particular visit.

Focus group 1

All participants thought it would be useful if technology could retrieve information about patients’ social well-being, such as whether they are socializing and leaving the house and their physical well-being such as their diet, home temperature, and physical activity.

…cause they could be isolated and not communicating outside of their home environment, and we may not know that. Similarly, you know, about not accessing food.

Focus group 6

All participants were particularly concerned about receiving detailed information about older patients’ falls; this information could be captured by wearable technologies or video systems. Information about falls is important when deciding if extra resources are needed or if someone should no longer be living on their own. Objective evidence would often be useful rather than relying on reports from clients or their family, who often differ in their perspectives.

…Knowing how often someone had fallen would be evidence. Quite often we have to put quite an argument to the Ministry of Health for funding for things and if you said subjectively, I am sure that this person has bad balance and is at risk of falling, but if you had a concrete “they've fallen 10 times in the last 6 weeks” then that's 10 falls.

Focus group 1

…Actually a fall is a good event to focus on because even though they wear their alarms, they don’t often push them, elderly people, so, you know, it’s good.

Focus group 7

The primary care doctors were also interested in the possibilities of using technology to improve the telemonitoring of patients’ parameters such as blood pressure, blood glucose levels, peak flow readings, and weight.

…I suppose that’s already happening, I know [xxx] has got quite a few patients that have got the blood pressure monitoring that they email him blood pressure results, the same with blood sugar, glucose results.

Focus group 5

However, they were concerned about being overwhelmed with data and were enthusiastic about the idea of information being preprocessed and filtered and only passed on when exceptions arise.

…You don’t want stuff pushed at you, you just want to see the exceptions, wow, something really strange is happening, yeah. So very much processed and filtered before it gets to you. You don’t want a daily update on everything.

Focus group 5

Furthermore, any new information should be seamlessly integrated into the existing systems.

…That's a really good point, cause we don't want too many databases we have to go into. It's a nightmare… you've got to consolidate in some way. Yeah, no absolutely.

Focus group 6

However, primary care teams were clear that they were unwilling to assume the additional responsibility of managing such systems.

…Why does it always have to go back to a nurse or a doctor? …we’re already busy and overwhelmed with our workloads that we may not be the right people.

Focus group 5

### Theme 3: Barriers to Uptake

Participants were optimistic about technology retrieving information and were clear about the potential barriers to uptake. This theme encompasses 3 subthemes: usability, cost, and security and privacy. Participants were concerned about software complexity and usability issues associated with aging, such as dexterity and cognitive decline. Many participants indicated that the training required for older people would be substantial and that their desire to learn to use technology would be mixed.

…I give them the computer cord (to connect the glucose monitor to their computer) to use download express software, to download their blood glucose monitor and email it to me...I haven't had any success...The program is quite convoluted for them...younger techie people just pick it up.

Focus group 6

…With ManageMyHealth [patient portal] they're keen to engage. But then it's a struggle. And quite often we'll just take them out the back and actually do it all for them.

Focus group 6

Participants considered cost to be another barrier and discussed how it is already a barrier for their uptake of health products.

…When I talk to patients about personal alarms, it always comes down to the cost and “how much are they? I can't afford that”… so cost, I would say, would be a huge thing, especially for the elderly.

Focus group 2

…The majority of the elderly have not got that extra $15 a week to pay for a blister pack (medicine sachet packaging)… $60 a month—that’s whether you’re going to keep the heater on.

Focus group 5

Ensuring privacy and data security was considered to be a requirement to protect patients. Some participants felt that clients would have concerns about privacy and data security, although this barrier did not present as strongly compared to usability and cost.

…They get quite protective of their privacy because as they get older they feel they are losing more and more.

Focus group 1

…Any piece of equipment or technology, privacy is a big thing.

Focus group 1

It was clear from the participants that sharing information with informal support networks is common, although some participants indicated the need to obtain informed consent.

…We try and help to facilitate that everybody is on the same page and know all the information.

Focus group 3

…Well, we have to gain the patient's consent for information to be shared with family. And we have to be very cautious about what medical information we share.

Focus group 3

Respondents had concerns about sharing information with family and how the information might be used as there may be conflicts within the family, for example, about what are the most appropriate arrangements for care.

…We might also talk to them if we have concerns about the patient’s safety… it’s a fine line between breaching confidentiality and what is appropriate to actually divulge to a support person.

Focus group 4

…Are we monitoring for the right reasons? Is that person being monitored for the health benefits or are they being monitored because... you know there is the safety side of it but there is also the dilemma of what the other dimensions of the family are wanting. Is it exactly what your client wants?

Focus group 1

## Discussion

### Overview

The work reported in this paper is part of the requirements-gathering phase of an exploratory project, which investigates how technologies that connect older people to their informal and formal support networks could assist aging in place and enhance older people’s health and well-being. Seven focus groups consisting of 44 health care professionals considered their information needs and the ways that technology could help meet some of those needs. The limited size of the study means that care should be taken not to overgeneralize the conclusions, but several common themes emerged within the findings.

Although the use of telemonitoring and smart home technologies to support older people has been proposed since many years, there has been very limited uptake [21,22], especially within the public health care systems [23,24]. However, many older people and their families are recognizing the benefits that these monitoring technologies can provide in terms of safety and peace of mind for all concerned, and commercial organizations are starting to provide related products and services [25,26]. Such organizations tend to work on a fee-for-service model, where the user pays to have their information collected, monitored, and acted upon as necessary in a limited number of ways. These systems tend to be closed cloud-based ecosystems with the information remaining with the service provider.

For monitoring systems to reach their full potential to improve the well-being of all older people, they must be expanded and integrated into the wider health care system. This raises the information governance issues of how the data and information collected by smart home technologies and other monitoring technologies can be effectively integrated with other systems and made available to the health care workforce.

### Access to Technology and Systems

The first major theme to emerge from the focus groups was that there remains a lack of integration between current information systems, especially between primary care and secondary care institutions. Such a lack of integration has been identified as a major problem in previous studies [27-29]. Secondary care also has multiple computer-based systems as well as paper-based notes, which inhibits the easy sharing of appropriate information between health care professionals. Furthermore, many other government departments, nongovernmental organizations, and private service providers often possess useful information about patients, which is not shared with the health service. Participants were frustrated by their inadequate access to information, as practitioners described having little information about patients before visits. This is not only a safety concern, but it is also well-established that information access can impact decision-making and the quality of care and efficiency [30]. This is a particular issue in aged care, as older people are more likely to have chronic conditions and disabilities [3]. Although the New Zealand Ministry of Health is working toward a more integrated information ecosystem [31], the current information system fragmentation does not place the patient at the center of health care delivery. This disconnected information architecture means that currently, there is no clear way for sensor data from telemonitoring and smart home devices to be integrated with other patient information. In some ways, primary care might seem to be the most appropriate site for storing the information since primary care clinics are seen as the site of ongoing, long-term relationships with the patients [32,33]; however, the participants from primary care organizations in this study were unwilling to take on the responsibility. Ultimately, it might be appropriate to create a new organization to take on the responsibility for the implementation and governance of systems to collect, process, and disseminate this monitoring information to all interested parties in the health care sector and to informal support networks.

### Collecting and Sharing of Information

Research into telemonitoring and smart homes has identified many ways to collect information that can be useful for various health care providers. However, focus group participants emphasized that they were concerned that they should not be overloaded with information. They wanted to be able to tailor the delivery of information for each individual patient and to have it preprocessed so that only exceptional situations were identified and flagged. The feasibility of processing information on exceptions with blood pressure recordings by telemedicine systems has been demonstrated in previous research [34,35] and similar techniques should be integrated into future developments. Participants also wanted the information to be integrated with their existing patient management system so that they only needed to access 1 system.

### Barriers to Uptake

The findings also reaffirm several user requirements deduced from interviews previously undertaken with older people and informal support networks [14]. Some issues addressed by these requirements correspond to barriers already identified in health informatics research. For example, cost is an issue for many older people [21,36], and governments should undertake detailed cost-benefit analyses of these systems to determine what level of public funding is appropriate. Usability is another barrier [21,36]; therefore, the technology should be designed for ease-of-use. A direct user interface may not be suitable for some older people, particularly for those living with cognitive decline. A number of systems have been developed that use motion sensor technologies to passively monitor the home environment and send alerts when certain out-of-the-ordinary events occur [37,38], and future developments should consider the integration of such systems.

Concerns about privacy and the desire to control the distribution of information were also seen as barriers to uptake, which have also been widely recognized in other studies [23,39,40]. The information needs of informal support networks have been largely neglected in previous research [21], even though they are often essential for enabling aging in place [21], and inadequate information access has been shown to negatively impact their ability to assist older people [10]. An important finding was that participants indicated that older people’s support networks are frequently a considerable part of information exchange, both for retrieving and providing information. However, this is generally undertaken through an informal means, rather than through a specific technological solution. This is an example of technology failing to address the realities of supporting aging in place as a collaborative and information-critical activity. However, providing access to information from home-monitoring systems to an older person’s informal support network will require much stricter controls to ensure privacy and confidentiality than is necessary for a system that is restricted to health care personnel.

### Limitations

The findings of this study are limited to the 44 participants recruited and the organizational settings in which they work. Reflective of the nature of qualitative enquiry [41], the participant recruitment was not drawn from a random sample of subjects, but it rather comprised individuals and organizations in the Manawatū region in New Zealand that actively volunteered to be involved in the study. This resulted in the participant number varying across the focus group sessions and the participants only being employed at general health practices and hospital departments, although they represented a range of health professionals. There are also limitations that are inherent to the nature of conducting focus groups, which can involve complex social dynamics, especially within organizations; therefore, the findings should be considered as a product of the groups and not as individuals [42]. Care should be taken when generalizing the findings drawn from the study beyond the study locations, and future research examining this topic should certainly take a wider scope.

### Conclusion

The 44 participants working with community-dwelling older people in this study want more information about their patients’ well-being within their homes, thereby demonstrating a potential for home monitoring and information communication technologies to connect older people to their formal support networks. This would not only assist health practitioners but would also support aging in place, which is socially and economically beneficial, as life expectancy continues to rise [3]. Our analysis of the discussions within the focus groups highlights the importance and the complexity of management and governance of systems to collect and disseminate such information. Issues that will need to be considered in the future are as follows.

1. Where will the data be stored and who will be responsible for its governance?
2. Who should pay for the technology and the ongoing running costs?
3. How will data be processed before it is presented?
4. How will the information be integrated into other systems used by health care providers?
5. To what extent should the information be released to informal support networks?

Further research into the requirements of all stakeholder groups is required to address these issues, which need to be resolved before the true potential of these technologies can be realized in practice.
